# The role of DMARDs in reducing the immunogenicity of TNF inhibitors in chronic inflammatory diseases

**DOI:** 10.1093/rheumatology/ket260

**Published:** 2013-08-14

**Authors:** Meghna Jani, Anne Barton, Richard B. Warren, Christopher E. M. Griffiths, Hector Chinoy

**Affiliations:** ^1^Arthritis Research UK Epidemiology Unit, Manchester Academic Health Science Centre, University of Manchester, ^2^NIHR Manchester Musculoskeletal Biomedical Research Unit, Central Manchester Foundation Trust, Manchester Academic Health Science Centre and ^3^The Dermatology Centre, Salford Royal NHS Foundation Trust, Manchester Academic Health Science Centre, University of Manchester, Manchester, UK

**Keywords:** immunogenicity, anti-drug antibodies, methotrexate, disease-modifying anti-rheumatic drugs, azathioprine, anti-TNFs, biologics

## Abstract

The management of RA, SpA, psoriasis and inflammatory bowel disease has significantly improved over the last decade with the addition of tumour necrosis factor inhibitors (anti-TNFs) to the therapeutic armamentarium. Immunogenicity in response to monoclonal antibody therapies (anti-drug antibodies) may give rise to low serum drug levels, loss of therapeutic response, poor drug survival and/or adverse events such as infusion reactions. To combat these, the use of concomitant MTX may attenuate the frequency of anti-drug antibodies in RA, SpA and Crohn’s disease. Although a similar effect to methotrexate has been observed with AZA usage in the management of Crohn’s disease, there is insufficient evidence to suggest that other DMARDs impact immunogenicity. In this article we review the evidence to date on the effect of immunomodulatory therapy when co-administered with anti-TNFs. We also discuss whether such a strategy should be employed in SpA and psoriasis, and if optimization of the MTX dose could improve biologic drug survival and thereby benefit disease management.

## Introduction

TNF-α inhibitors (anti-TNFs) have transformed the treatment paradigm of autoimmune diseases such as RA, psoriasis, PsA, AS and IBD, where standard systemic agents have failed. As revolutionary as these therapies are, not all patients respond favourably and response rates are of the order of 40–70% after 3–4 months of treatment, depending on indication. This lack of initial efficacy is known as primary non-response. However, in a significant proportion of patients, anti-TNFs lose efficacy over time, with ensuing disease relapse known as secondary non-response.

### Implications of immunogenicity

Immunogenicity refers to the ability of protein drugs to provoke an immune response. The immune system can detect small differences in the three-dimensional structure between an introduced foreign molecule and a native protein, leading to the production of anti-drug antibodies (ADAbs) [1]. Recent evidence has demonstrated that ADAb formation, particularly in response to monoclonal antibodies such as infliximab and adalimumab, is an important mechanism underlying therapeutic failure and loss of response over time in RA [2–4], SpA [5] and Crohn’s disease [6]. A similar effect correlating drug response and ADAb formation has not been observed with etanercept, which is thought to be less immunogenic, as discussed below, although other mechanisms may play a role where loss of efficacy occurs. Antibodies to a drug may be either binding or neutralizing, and can lead to a loss of response by altering the pharmacokinetics, resulting in subtherapeutic levels, or decreasing efficacy by neutralizing the active component of the molecule [7]. In some cases, immune complex–mediated adverse events such as serum sickness, Arthus reactions, bronchospasm, infusion reactions and venous/arterial thromboembolic events have also been reported in association with immunogenicity [8–11].

### Factors affecting immunogenicity

Both the European Medicine Agency and the Food and Drug Administration have published guidelines relating to unwanted immunogenicity of monoclonal antibodies for *in vivo* clinical use, outlining the mandatory assessment of immunogenicity for the approval of biopharmaceuticals [12, 13]. The detection of ADAbs is dependent on factors including the timing of the sample taken relative to dosing, duration of treatment and, importantly, the assay used (Table 1). ELISAs have mostly been utilized for testing because of their low cost and high throughput. However, ELISA-based detection methods are more prone to drug interference and do not detect IgG4 ADAbs, which have a greater potential for neutralization [7, 14]. RIA has the ability to detect IgG4 antibodies, is less prone to drug/rheumatoid factor interference and has been used successfully in more recent prospective studies (Table 2), but is more expensive and requires the use of radioisotopes.

**Table 1:** Factors affecting immunogenicity

Detection of anti-drug antibodies	Drug-related factors	Individual characteristics	Treatment-related factors
Type of assay	Contaminants in the formulation process	Immunocompetence of the patient	Dose and frequency of drug
Timing of blood sample	Structural properties	Genetic predisposition	Route of administration
Duration of treatment	Sequence variation/murine components	Unknown factors	Use of concomitant immunomodulatory drugs
	Target binding ability		
	T cell epitopes

**Table 2:** Effect of DMARDs on immunogenicity in response to anti-TNF therapy in RA, PsA and AS

Author	Disease	Anti-TNF	*n*	Follow-up, months	DMARD	Mean DMARD dose^a^ (patients without ADAb *vs* with)	Assay	Overall ADAb frequency, %	ADAb % DMARD group	ADAb % non-DMARD group	*P*-value	Comments
Maini *et al.* [16]	RA	IFX	101	6	MTX	7.5 mg/week (NS)	ELISA	17.4	0–15	7–53	NA	Immunogenicity assessed as part of a double-blind RCT evaluating safety, efficacy and pharmacokinetics
Bendtzen *et al.* [17]	RA	IFX	106	18	MTX, SZ, AZA, CYP, HCQ, pred	NA	RIA	44	40 (MTX only)	50 (MTX only)	NA	Concomitant MTX lowered levels of ADAbs unlike other DMARDs or pred
Wolbink *et al.* [67]	RA	IFX	51	12	MTX	15 mg/week	RIA	43	NA	NA	NA	Baseline characteristics of patients with and without ADAbs, including mean dose of MTX were similar. None of the three patients on AZA developed ADAbs.
					AZA	NA						
					CYP	NA						
Pascual-Salcedo *et al.* [4]	RA	IFX	85	6	MTX	15 mg/week	ELISA	32.9	32	37	NS (*P* = 0.77)	Use of MTX was associated with lower levels of ADAbs. Pred prescribed in 74% of patients, other DMARDs in 18%: association with ADAbs not reported.
					Pred	NA						
Bartelds *et al.* [18]	RA	ADA	121	6	MTX	19.4 mg/week (17.4 *vs* 19.7)	RIA	17	12	38	0.003	Concomitant MTX use was lower in the group with ADAbs (52%) than in the group without antibodies (84%).
Bartelds *et al.* [19]	RA	ADA	235	6	MTX	20 mg/week (18 *vs* 20)	RIA	20	NA	NA	<0.0001	Of all patients without ADAbs to adalimumab, 89% used concomitant MTX treatment compared with 54% of the patients with anti-adalimumab antibodies (*P* < 0.0001).
					Pred	7.5 mg/day (10 *vs* 5)						
Bartelds *et al.* [2]; Krieckaert *et al.* [20]	RA	ADA	232	36	MTX	Median dose 25 mg/week (25 *vs* 18)	RIA	28	12–35	Up to 50	<0.001	Dose-response relationship seen with increasing MTX dose and immunogenicity. Pred or other DMARDs did not show an association with reducing ADAb formation.
					Pred	Median dose 7.5 mg/day (5 *vs* 7.5)						
					SZ/HCQ	NA						
Emery *et al.* [68]	RA	GOL	315	6	MTX	19 mg/week	ELISA	6.3	1.9–3.7	13.5	NA	Monotherapy patients had a higher incidence of ADAbs at 13.5% compared with those receiving MTX with either golimumab 50 mg (3.7%) or golimumab 100 mg (1.9%).
Kavanaugh *et al.* [33]	PsA	IFX	200	16.4	MTX	16.7 mg/week	NA	15.4	3.6	26.1	NA	Phase III RCT evaluating safety and efficacy in PsA patients on IFX. Oral glucocorticoids used in 15%; effect on ADAb not reported.
					Pred	NA						
Ducourau *et al.* [34]	SpA	IFX	91	36+	MTX	NA	ELISA	19	0	32	0.03	17 with RA and 91 with SpA were evaluated. The median time to ADAb detection after initiation of infliximab was 3.7 months (1.7–26.0 months).
					Pred	NA			2	12	NS (0.8)	
Plasencia *et al.* [5]	SpA	IFX	94	84+	MTX	15 mg/week	ELISA	25.5	11	34	0.011	MTX was significantly associated with a reduction in ADAbs. Steroid use was present in 41.8% and other DMARDs used in 26.6%, however, no data were reported on dose/effect on ADAbs.
					Corticosteroid treatment	NA						
					Other DMARDs	NA						

^a^Unless otherwise specified.

ADA, adalimumab; CYP, ciclosporin; GOL, golimumab; IFX, infliximab; NA, not analysed; NS, not significant; pred, prednisolone.

The development of ADAbs can be influenced by drug-related factors [1], individual patient characteristics, including immunocompetence and genetic predisposition [15], as well as treatment-related factors (Table 1). One of the few externally modifiable factors on immunogenicity from the clinician perspective is the drug dosage/frequency and co-administration of immunomodulators. Concomitant use of certain DMARDs such as MTX may maintain efficacy and prolong drug survival by reducing ADAb formation to anti-TNFs. DMARDs may thus circumvent the unfavourable consequences of immunogenicity on both the efficacy of monoclonal antibody–based biologics and possibly immune complex–mediated adverse events. An issue of great interest in decreasing immunogenicity in both AS and psoriasis is the potential role of concomitant MTX, which is not routinely co-prescribed in these conditions. In this review we discuss the available evidence to date on the influence of concomitant DMARDs on the immunogenicity of anti-TNFs in chronic inflammatory conditions.

## Rheumatoid arthritis

### Monoclonal anti-TNFs

#### Infliximab

**Fig. 1:**
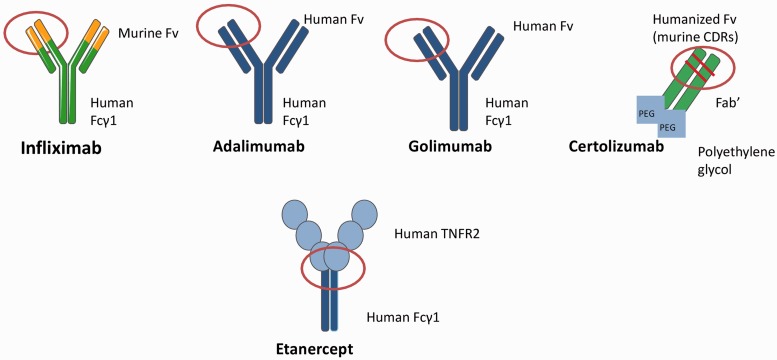
Molecular structure of anti-TNF drugs with potential immunogenic sites.

Infliximab is a chimeric protein containing 25% mouse-derived amino acids and 75% human-derived amino acids (Fig. 1). The variable murine region of infliximab is thought to be the antigenic component that induces the formation of human anti-chimeric antibodies. In a number of studies, the use of concomitant MTX appears to reduce the immunogenicity of infliximab (Table 2).

In 1998 Maini *et al*. [16] first investigated whether MTX could reduce the immunogenicity of infliximab in RA. This 26-week, double-blind, placebo-controlled, multicentre trial also evaluated the efficacy, pharmacokinetics and safety of infliximab by randomizing 101 patients into seven groups of 10–15 patients each, given alone or in combination with MTX, with different infliximab dosing regimens. The development of antibodies was inversely associated with infliximab dose (53%, 21% and 7% in patients receiving 1, 3 and 10 mg/kg monotherapy, respectively), and the use of concomitant MTX at a dose of 7.5 mg/week greatly diminished the appearance of ADAbs, with incidence rates of 15%, 7% and 0% at the three dose levels. Infliximab monotherapy at the lowest dose of 1 mg/kg induced the highest incidence of ADAbs, where patients in this group became unresponsive to repeated infusions of infliximab at 2.6 weeks. However, co-administration of MTX, even with the lowest dose of infliximab, appeared to be synergistic, prolonging the duration of the 20% Paulus criteria for response in >60% of patients to a median of 16.5 weeks (*P* < 0.001 *vs* placebo; *P* = 0.006 *vs* no MTX) and 50% response to 12.2 weeks (*P* < 0.001 *vs* placebo; *P* = 0.002 *vs* no MTX). The authors proposed that MTX virtually abolished ADAb responses when used with a higher dose of infliximab, possibly due to maintenance of higher circulating drug levels.

In a study of infliximab-treated RA patients, Bendtzen *et al*. [17] found that at 6 months, ADAb-positive patients receiving MTX had lower antibody levels than those not receiving MTX (11% *vs* 5%, *P* = 0.037). Concomitant use of other DMARDs such as SSZ, AZA, ciclosporin, HCQ or prednisolone did not significantly affect antibody levels. This observation was also noted in a Spanish study that used a sandwich ELISA to evaluate the effect of long-term immunogenicity in a cohort of 85 infliximab-treated RA patients. In this study, concomitant MTX use was not significantly associated with a lower proportion of ADAbs, however, those receiving both infliximab and MTX tended towards lower levels of anti-infliximab antibodies (*P* = 0.073) and longer survival (*P* = 0.015) on treatment [4]. The development of immunogenicity in this study was strongly linked to infusion reactions, a need to increase the frequency of dosing regimens due to poor response and shorter median drug survival compared with patients without ADAbs (4.15 *vs* 8.89 years, *P* = 0.0006).

#### Adalimumab

Similar findings to the infliximab studies have been reported by Bartelds *et al.* [2, 18, 19] using RIA in RA patients treated with adalimumab. Although adalimumab is a fully human antibody, there still remains the potential to induce human anti-human antibodies. In a prospective cohort study over 28 weeks, the anti-adalimumab antibodies developed in 17% of RA patients and were associated with a reduced improvement in disease activity (mean ΔDAS28, ADAb positive 0.65 ± 1.35 *vs* ADAb negative 1.70 ± 1.35; *P* = 0.001). The use of concomitant MTX was related to a lower rate of antibody development than adalimumab monotherapy (12% *vs* 38%) [18]. Immunogenicity was subsequently increased in the context of switching from infliximab to adalimumab due to non-response [19]. Patients who developed prior anti-infliximab antibodies (33/52 switchers, 63%) more often developed anti-adalimumab antibodies compared to anti-TNF naive patients and consequently were less likely to respond to adalimumab compared with patients who did not develop anti-adalimumab antibodies. However, of all the patients without ADAbs to adalimumab, 89% used concomitant MTX compared with only 54% of the patients with anti-adalimumab antibodies (*P* < 0.0001). The same group also evaluated the impact of immunogenicity in 272 RA patients treated long term with adalimumab, where 28% of patients developed ADAbs over 3 years, the majority (67%) in the first 6 months. The development of ADAb formation was significantly associated with poor rates of remission [DAS28 < 2.6; hazard ratio (HR) 7.1; 95% CI 2.1, 23.4; *P* < 0.001], reduced likelihood of minimal disease activity (DAS28 < 3.2; HR 3.6; 95% CI 1.8, 7.2; *P* < 0.001), as well as higher rates of drug discontinuation due to treatment failure (38% *vs* 14%; HR 3.0; 95% CI 1.6–5.5; *P* < 0.001) [2]. Patients who developed anti-adalimumab antibodies during the 3 years were much less likely to be on concomitant MTX at baseline (52% *vs* 82%; *P* < 0.001) and on a lower mean dose (18 *vs* 25 mg/week; *P* < 0.005). The use of other concomitant DMARDs such as SSZ and/or HCQ was not associated with such an effect, however, these drugs were used much less frequently (7% of the total RA cohort). Furthermore, prednisolone use or dose was not significantly different in patients who developed ADAbs to those who did not (36%, median dose 7.5 mg/day *vs* 33%, median dose 5 mg/day, respectively).

The relationship between immunogenicity and MTX was further explored by Krieckaert *et al*. [20] in the above group of patients, who demonstrated a clear dose-dependent relationship with MTX and a reduction in ADAb formation. RA patients in the adalimumab cohort (*n* = 272) were stratified according to the baseline MTX dose: no concomitant MTX (*n* = 70), low dose (5–10 mg/week, *n* = 40), intermediate dose (12.5–20 mg/week, *n* = 54) or high dose (≥22.5 mg/week, *n* = 108). Patients using MTX developed ADAbs less often compared with patients who were untreated [odds ratio (OR) 0.20, 95% CI 0.12, 0.34; *P* < 0.001]. As the dose of MTX increased as stratified in the four groups, this was inversely proportional to the percentage of patients developing ADAbs; the ≥22.5 mg/week group contained the lowest proportion of patients developing immunogenicity.

### Newer monoclonal antibodies

Fewer studies have investigated the relationship between clinical response and immunogenicity with the newer monoclonal antibodies golimumab and certolizumab pegol. Golimumab is a fully human IgG molecule, while certolizumab pegol is a humanized Fab fragment attached to polyethylene glycol (PEG) and contains amino acid sequences in the complementarity-determining regions derived from a mouse (Fig. 1). The addition of PEG increases the half-life of certolizumab pegol and may reduce the immunogenicity of some biopharmaceutical proteins [1, 21]. Randomized controlled trials (RCTs) report ADAbs in a small proportion of patients on golimumab and certolizumab, however, the numbers of ADAb-positive patients were insufficient to determine a clear association with impaired therapeutic response [22–26]. A reduction in immunogenicity with concomitant MTX has been observed in RA patients on golimumab [27], where 13.5% of those on monotherapy developed ADAbs compared with only 1.9% in patients on an optimal dose of golimumab plus MTX. Although most RCTs in RA detecting ADAbs to certolizumab pegol did not find an association with treatment response, it should be noted that a monotherapy trial did demonstrate a correlation [27]. In the FAST4WARD study, which randomized patients to certolizumab pegol monotherapy or placebo, 8.1% of subjects developed neutralizing antibodies (assessed by a cell-based assay) to certolizumab at 24 weeks. The ACR20 response was reduced by an estimated 5% in patients who developed ADAbs. Further prospective observational studies are required to fully assess the immunogenic potential of golimumab and certolizumab in relation to drug response and survival.

## SpA and psoriasis

An important question is whether MTX should be prescribed in combination with biologic therapy in patients with AS, where DMARDs are not routinely prescribed for axial disease, and in psoriasis, where MTX is often discontinued before commencing biologic therapy. Concomitant use of MTX may improve drug survival, reduce immunogenicity and prevent secondary inefficacy, which is of particular significance in AS and psoriasis, where, compared with RA, there are fewer classes of biologics to switch to in the event of treatment failure.

### MTX and AS

The efficacy of MTX used in conjunction with infliximab has previously been evaluated in AS outside the context of immunogenicity with conflicting results [28–31]. A 30 week open label study in 19 patients with active AS assessed whether the addition of MTX to infliximab could increase therapeutic efficacy [29]. The nine patients who were on concomitant MTX (dose 7.5 mg/week) achieved a significantly better BASDAI 50 response compared with monotherapy patients; however, patients included in the combination therapy group were younger and had shorter disease duration at baseline, factors both known to affect drug response, making these results difficult to extrapolate. Breban *et al*. [28] evaluated concomitant MTX with infliximab in a subgroup of AS patients by using an on-demand strategy in which patients received an infusion only if they relapsed. Continuous treatment of patients with infliximab was clearly superior to an on-demand regime, which may be due to the development of ADAbs (although these were not measured), a phenomenon also seen in RA patients who had interrupted adalimumab treatment schedules [32]. A trend towards fewer reactions to infusions in the group receiving MTX was also seen, although these results were not statistically significant. The use of MTX in AS patients did not lead to a significant increase in adverse events when compared with the monotherapy group—a potential concern when evaluating the risks and benefits in this group of patients who are not routinely co-administered DMARDs.

### SpA and immunogenicity

In the IMPACT2 trial, which evaluated the safety and efficacy of infliximab in PsA, 47% of patients were on concomitant MTX (mean dose 16 mg/week) [33]. By Week 66, while only 3.6% of patients receiving MTX at baseline were positive for antibodies to infliximab, 26.1% of those not receiving MTX at baseline tested positive. ADAbs were inversely correlated to the ACR20 response and ADAb formation conferred a 3.5-fold increase in mild to moderate infusion reactions. Ducourau *et al*. [34] evaluated 91 SpA patients on infliximab long term and also found a higher rate of ADAbs in patients not on concomitant MTX [ADAb formation, no concomitant MTX 0/14 (0%) *vs* concomitant MTX 25/77 (32%); *P* = 0.03]. A potential weakness of this study is that the SpA subtype was not clearly defined, given the differences in baseline MTX use between AS and PsA and likely differences in immunogenic potential between the two groups. No differences in immunogenicity were observed in patients who received concomitant prednisolone in either study.

The use of MTX has also been associated with a lower incidence of ADAbs to golimumab in both PsA [35, 36] and AS [37] in the context of RCTs. The GO-REVEAL trial of golimumab in PsA reported a low incidence of ADAbs (4.6% at 6 months), however, these were present in none of the patients on MTX [35]. A similar incidence of ADAbs was reported at 12 months (4.9%) [36], with the majority of patients with antibodies to golimumab (18/19) not on concomitant MTX. The GO-RAISE study [37] assessed outcomes in AS patients on golimumab at 6 months, none of whom were on MTX, reporting a similar ADAb frequency (4.1%). Most recently Plasencia *et al*. [5] evaluated the long-term effect of immunogenicity in 94 SpA patients on infliximab using a bridging ELISA (50 AS, 12 undifferentiated SpA, 22 PsA and 10 SpA associated with IBD). ADAb formation was present in 25.5% of patients overall. Patients with ADAbs to infliximab had significantly higher AS disease activity scores than those without antibodies at all time points (6 months, 1 year and >4 years), with lower drug levels and poorer drug survival (median survival 4.25 *vs* 8.85 years, *P* < 0.001). A total of 47 patients (50%) were on concomitant MTX at a mean dose of 15 ± 4.96 mg/week during the study, however, only 38% were on MTX prior to starting anti-TNF treatment. ADAbs to infliximab developed more frequently in patients not on MTX [no concomitant MTX, 20/58 (34%) *vs* concomitant MTX 4/36 (11%), *P* = 0.011]. Concomitant MTX was also associated with delayed development of ADAbs in the patients who did develop immunogenicity, which may partly explain why some shorter duration studies failed to demonstrate an effect of MTX on infliximab in AS [30, 31].

### Psoriasis and use of concomitant MTX

In psoriasis, studies have shown a trend in favour of MTX use to reduce immunogenicity in response to anti-TNFs, however, the sample sizes had limited power to detect statistically significant differences [38, 39]. Lecluse *et al*. [38] assessed immunogenicity in 29 patients on adalimumab over 26 weeks, of whom 45% developed ADAbs. Although only 10% of patients were on concomitant MTX, none of these patients developed drug antibodies to adalimumab. Interestingly, Adisen *et al*. [39] reported a reduction in the psoriasis area severity index after the introduction of MTX at doses between 5 and 15 mg/week in patients who had already developed ADAb formation in response to infliximab after 8 weeks (four of five patients). The addition of MTX, even after the development of ADAbs, could potentially provide an alternative treatment strategy in those patients who develop secondary non-response to monoclonal drugs and ADAbs measured through pharmacological monitoring in conditions where biologics are traditionally administered as monotherapy. Further work is needed to fully evaluate this effect, with larger prospective studies required to assess the role of co-administration of MTX with biologic therapy on immunogenicity and drug survival in patients with psoriasis.

## Etanercept

The soluble dimeric fusion protein etanercept is considerably less immunogenic than monoclonal antibodies [40, 41]. Etanercept is also administered more frequently than other biologics, possibly creating more drug interference in ADAb detection and more constant drug levels. The junction between the two receptors linked to the Fc portion of IgG1 comprises a murine sequence and may therefore have some immunogenic potential (Fig. 1). Most studies to date, however, have either failed to detect ADAbs to etanercept or have detected them at lower levels compared with monoclonals, the highest level detected being 18% in psoriasis at 12 weeks [42]. In cases where ADAbs were detectable, their presence did not correlate with either drug levels, adverse reactions or clinical response in RA [43–46], AS [47], PsA [48] or psoriasis [42, 49–51]. This suggests the possibility of binding antibodies (that do not neutralize the effect of the drug) or false positive results, as in the majority of studies ELISAs of low specificity were used. ADAbs to etanercept were not detected even when more sensitive assay techniques such as RIA were employed in RA [40] and AS [47]. Thus the effect of concomitant DMARD use on anti-etanercept antibodies has not been assessed. Those RA patients who do not respond to etanercept may still have lower serum drug concentrations compared with responding patients, despite the lack of detection of antibodies using RIA [40]. This may support a strategy to increase the frequency or dose of the drug in compliant patients not achieving adequate response.

## IBD

In Crohn’s disease, the use of concomitant immunomodulatory therapy has been associated with a reduction in immunogenicity in a number of studies of infliximab [6, 52–58]. Corticosteroids, when given in the form of intravenous hydrocortisone pre-treatment, have been reported to reduce anti-infliximab antibody concentrations, but not their formation [52]. Baert *et al*. [6] evaluated 125 patients with refractory luminal or fistulizing Crohn’s disease who were treated with infliximab infusions on demand over a mean period of 10 months. Antibodies to infliximab were detected in 61% of patients by their fifth infusion, and were associated with infusion reactions as well as reduced response to treatment. In contrast, the use of concomitant immunosuppressants (45% AZA/mercaptopurine, 2% MTX, 40% mesalazine and 42% corticosteroids) was associated with a lower incidence of ADAbs (43% *vs* 75%, *P* < 0.01). The beneficial effect of AZA in conjunction with infliximab on ADAb formation was also demonstrated in a large RCT assessing 508 patients assigned to receive infliximab monotherapy (5 mg/kg), AZA alone (2.5 mg/kg) or a combination of infliximab and AZA over 6 months, although the assay used for detection was not described [56]. Steenholdt *et al*. [58] reported on a cohort of 106 patients consisting of both ulcerative colitis and Crohn’s disease patients treated with infliximab. ADAb formation was significantly associated with a loss of response and low drug trough levels. As measured by RIA, the frequency of ADAb formation in those who did not receive concomitant DMARDs was significantly higher than those receiving AZA, 6-mercaptopurine, or MTX [no concomitant DMARDs, 16/32 (50%) *vs* concomitant DMARD use, 19/73 (26%), OR 2.8, 95% CI 1.2, 6.8, *P* = 0.02).

To assess whether antibodies to adalimumab affect treatment response in patients with Crohn’s disease treated with infliximab, a small cohort of 30 patients was evaluated over a mean duration of 318 days (range 83–632 days) [59]. The presence of ADAb formation in 17% of patients, as measured using RIA, was significantly associated with treatment non-response. Concomitant treatment was used in 13 patients (4 patients on AZA, MTX and corticosteroids each, 1 on mercaptopurine), of which only 1 patient (7.7%) developed ADAbs, compared with 20% of patients on monotherapy; however, this was not statistically significant, probably due to low power to detect a difference.

To investigate which drug, MTX or AZA, was most effective at reducing ADAb formation, a cohort of 174 Crohn’s disease patients on infliximab was studied [54]. Patients were stratified into three groups: 50 patients on concomitant MTX (15 mg/week), 65 patients on AZA (2–2.5 mg/kg) and 59 patients on infliximab monotherapy. The concomitant use of AZA or MTX was associated with a significantly lower incidence of ADAb compared with infliximab monotherapy (46% *vs* 73%; *P* = 0.001). However, no significant differences were seen between the development of ADAbs in the MTX group (44%) compared with the AZA group (48%). Immunogenicity was associated with a shorter duration of response in patients on monotherapy as compared with patients taking concomitant AZA or MTX. Of interest is that the duration of treatment response was not influenced by AZA/MTX use when ADAbs were absent, further supporting the role of concomitant immunosuppressants in immunogenic modulation.

## Conclusion

A recent meta-analysis revealed that the use of immunosuppressants, primarily MTX, reduced the proportion of patients on infliximab and adalimumab with detectable ADAbs by about 41% (RR = 0.59, 95% CI 0.50, 0.70) [60]. However, this was dependent on the type of assay used: with RIA, concomitant immunosuppression reduced detectable ADAb by 64%, when ELISA methods were employed detectable ADAbs were reduced by 37%. The mechanism whereby MTX acts on the immune response has not been fully demonstrated, however, suppression of early T and B cell expansion may be responsible for modulation of the immune response [61]. Other researchers hypothesise that a synergistic effect of MTX with biologics may be responsible, as it reduces inflammation. Therefore, assuming MTX treated and untreated patients receive the same anti-TNF dose, a reduced level of TNF may consume less anti-TNF antibody, resulting in higher circulating drug levels, thereby accelerating ADAb clearance [10]. The polyglutamation of MTX has also been associated with an improved pharmacokinetic profile and lowered immunogenicity of infliximab [62]. It has been hypothesized that this may be due to its potent effect on aminoimidazole carboxamide ribonucleotide (AICAR) transformylase followed by purine biosynthesis inhibition and suppression of T cell clonality [62–64]. Functional work in this area may further elucidate the mechanisms underlying the influence of MTX on immunogenicity.

Although AZA (2–2.5 mg/kg/day) is beneficial in reducing ADAb formation in Crohn’s disease, this has not been reported in rheumatological conditions. No studies to date have investigated a reduction in ADAb formation with concomitant leflunomide and at present there is insufficient evidence to support the role of other DMARDs or prednisolone in reducing ADAb formation and thus improving drug survival. When added to anti-TNFs in the management of AS, RA or Crohn’s disease, the use of immunomodulators has not been associated with a significantly increased risk of serious adverse reactions [28, 65, 66] and may in fact reduce the rate of infusion reactions [65]. In SpA and psoriasis, although the addition of MTX to anti-TNFs does not improve the efficacy of anti-TNFs (unlike RA), in the event of ADAb formation, concomitant use at the onset of anti-TNF therapy could provide another therapeutic option for clinicians to optimize treatment response and avoid the adverse consequences of immunogenicity. Additional longitudinal data are required to assess appropriate dosing regimens and to ensure that the benefits of the additional drug outweigh the risks of further long-term immunosuppression. In the future, pharmacological monitoring for ADAbs followed by optimization of the MTX dose in putative RA patients who may have a genetic predisposition rendering them prone to immunogenicity may lead to anti-TNF dose reductions in those achieving remission. The potential for prolonging drug survival and preventing secondary non-response in patients on anti-TNFs would not only have significant cost implications, but clear benefits to the patient in providing a longer duration of disease-free remission in those with an initial good response to monoclonal-based therapies.

**Rheumatology key messages**

- Concomitant MTX reduces the immunogenicity of anti-TNFs in RA, Crohn’s disease and SpA.
- Optimization of the MTX dose in RA patients on anti-TNFs may prolong drug survival.
- Further research is required to assess if psoriasis or AS patients should use concomitant MTX with anti-TNFs.
